# Elevated level of inhibin-*α* subunit is pro-tumourigenic and pro-metastatic and associated with extracapsular spread in advanced prostate cancer

**DOI:** 10.1038/sj.bjc.6605089

**Published:** 2009-05-12

**Authors:** P Balanathan, E D Williams, H Wang, J S Pedersen, L G Horvath, M G Achen, S A Stacker, G P Risbridger

**Affiliations:** 1 Centre for Urological Research, Monash Institute of Medical Research, Monash University, Melbourne, Victoria 3168, Australia; 2 Centre for Cancer Research, Monash Institute of Medical Research, Monash University, Melbourne, Victoria 3168, Australia; 3 Tissupath Pty Ltd, 165 Burwood Road, Melbourne, Victoria 3122, Australia; 4 Sydney Cancer Centre, Missenden Road, Sydney, New South Wales 2050, Australia; 5 Cancer Research Program, Garvan Institute of Medical Research, 384 Victoria Street, Sydney, New South Wales 2010, Australia; 6 Ludwig Institute for Cancer Research, Royal Melbourne Hospital, Post Office Box 2008, Parkville, Victoria 3050, Australia

**Keywords:** inhibin-*α* subunit, prostate cancer, metastasis, androgen independent

## Abstract

The biological function of inhibin-*α* subunit (INH*α*) in prostate cancer (PCa) is currently unclear. A recent study associated elevated levels of INH*α* in PCa patients with a higher risk of recurrence. This prompted us to use clinical specimens and functional studies to investigate the pro-tumourigenic and pro-metastatic function of INH*α*. We conducted a cross-sectional study to determine a link between INH*α* expression and a number of clinicopathological parameters including Gleason score, surgical margin, extracapsular spread, lymph node status and vascular endothelial growth factor receptor-3 expression, which are well-established prognostic factors of PCa. In addition, using two human PCa cell lines (LNCaP and PC3) representing androgen-dependent and -independent PCa respectively, we investigated the biological function of elevated levels of INH*α* in advanced cancer. Elevated expression of INH*α* in primary PCa tissues showed a higher risk of PCa patients being positive for clinicopathological parameters outlined above. Over-expressing INH*α* in LNCaP and PC3 cells demonstrated two different and cell-type-specific responses. INH*α*-positive LNCaP demonstrated reduced tumour growth whereas INH*α*-positive PC3 cells demonstrated increased tumour growth and metastasis through the process of lymphangiogenesis. This study is the first to demonstrate a pro-tumourigenic and pro-metastatic function for INH*α* associated with androgen-independent stage of metastatic prostate disease. Our results also suggest that INH*α* expression in the primary prostate tumour can be used as a predictive factor for prognosis of PCa.

Inhibins A and B are members of the transforming growth factor-*β* (TGF*β*) superfamily. Inhibins are heterodimers of an 18 kDa *α*-subunit disulphide linked to one of two 13 kDa *β*-subunits (*β*A and *β*B) resulting in inhibin A (*αβ*A) and B (*αβ*B) respectively. Primarily of gonadal origin, inhibins regulate pituitary follicle-stimulating hormone secretion by feedback inhibition. In humans, women produce both inhibin A and inhibin B (Welt *et al*, 1999; Welt and Schneyer, 2001), whereas in adult men inhibin B is the primary form (Marchetti *et al*, 2003). Inhibins have also been shown to be expressed in adrenal cortex, pituitary and prostate (reviewed in Risbridger *et al*, 2001). Other endocrine and paracrine functions of inhibins involve regulating members of the TGF*β* superfamily, such as TGF*β* itself, activins and bone morphogenic protein (BMP), by competing for their receptors (Wiater and Vale, 2003; Farnworth *et al*, 2007). This antagonistic effect of inhibin is amplified in the presence of the inhibin receptor, TGF*β* receptor III (TGF*β*RIII), also known as betaglycan.

The first study that linked inhibin to reproductive cancers showed that serum inhibin increased in women with granulosa cell tumours of the ovary (Lappohn *et al*, 1989) and inhibin currently serves as a robust biomarker for this cancer. A direct biological function for inhibin in carcinogenesis was demonstrated by Matzuk *et al* (1992) when they created the inhibin-*α* subunit (INH*α*) knockout mouse. Both sexes developed gonadal sex-cord stromal tumours with high penetrance and developed tumours in their adrenal glands after castration, showing that INH*α* can act as a tumour suppressor. Subsequent mouse model studies revealed a complex network of interactions involving inhibin, activin and other modifiers in the development and progression of gonadal and adrenal tumours in INH*α*-deficient mice (reviewed in Risbridger *et al*, 2001). Although it is clear that total inhibin as a serum test has utility in the initial detection and prognosis of certain types of ovarian cancers, the biological function of inhibin in tumourigenesis is far from clear.

We have observed both up- and down-regulation of INH*α* expression in prostate cancer (PCa) tissues dependent on the stage of disease. Studies from our laboratory on metastatic PCa epithelial cell lines demonstrated that high concentrations (400 ng ml^−1^) of recombinant inhibin A inhibited growth of androgen-responsive LNCaP cells, but not androgen-independent DU145 cells, suggesting that the effect of inhibin may be dependent on cell phenotypes related to specific stages of PCa (McPherson *et al*, 1997). Furthermore, we have shown loss of heterozygosity or epigenetically regulated loss or down-regulation of INH*α* in PCa patient samples and LNCaP, DU145 and PC3 cell lines (Mellor *et al*, 1998; Schmitt *et al*, 2002; Balanathan *et al*, 2004). Collectively, these data suggest a tumour suppressive function for INH*α* in the prostate. In contrast, new insights into the role of INH*α* in the prostate came from a recent study using a large cohort of PCa patient tissues that showed that INH*α* was frequently over-expressed in high-grade PCa (Risbridger *et al*, 2004a, 2004b). The intensity of INH*α* immunoreactivity was associated with a higher risk of recurrence of PCa. These variable clinical and experimental observations provide equivocal evidence for a role of INH*α* in PCa. Thus it was proposed that like TGF*β* (Roberts and Wakefield, 2003), INH*α* has tumour suppressive activity in normal epithelial cells, which changes to tumour promoting in cancer cells (Ball *et al*, 2004; Risbridger *et al*, 2004a).

To date there is no proof that INH*α* can increase tumourigenesis and metastasis. Thus the primary aim of this study was to examine the pro-tumourigenic and pro-metastatic function of INH*α* in advanced PCa. Specifically, the expression profile of INH*α* and clinicopathological parameters were examined in primary PCa tissues including specimens from patients with organ-confined disease and those with metastasis to the lymph nodes. In addition, human metastatic PCa cell lines, LNCaP and PC3, were used in *in vitro* and *in vivo* functional studies to determine the biological function of elevated levels of INH*α* on migration, invasion, tumour growth and metastasis.

## Materials and methods

### Analysis of clinical material

#### Relationship between INH*α* expression and clinicopathological parameters in primary prostate adenocarcinomas

This study was conducted in accordance with Australian National Health and Medical Research Council (NHMRC) guidelines. Archival formalin-fixed paraffin-embedded tissue blocks were retrieved from 37 patients with prostate carcinoma who underwent radical prostatectomy. The clinicopathological characteristics, vascular endothelial growth factor-C (VEGF-C) expression, lymphatic vessel density (LVD) and lymph node status of this cohort have been described previously (Zeng *et al*, 2004, 2005). We conducted a cross-sectional study to determine whether INH*α* expression was associated with clinicopathological parameters (Gleason score, surgical margins, extracapsular spread, VEGF receptor-3 (VEGFR-3) expression and lymph nodes status) and/or linked to well-established prognostic factors in prostatic adenocarcinoma. The PO 12 antibody (kindly provided by Dr Nigel Groome) was used to determine the expression pattern of INH*α* in primary prostate tissues as previously described (Risbridger *et al*, 2004b). Each immunostained tissue section was assessed and staining intensity in benign epithelial, cancer (Gleason grade G3/G4) and stromal regions was scored from 0 to 4 with 0 representing negative staining and 4 representing very strong positive staining. The relative risk of PCa patients being positive for the respective parameters was determined.

#### Stable transfection of LNCaP and PC3 cell lines

LNCaP and PC3 were obtained from American Type Culture Collection (Rockville, MD, USA) and routinely cultured as described previously (Balanathan *et al*, 2004). Expression vectors pcDNA3.1 (empty vector, EV) and human *INHα* cDNA subcloned into pcDNA3.1 (pcDNA3.1 (INH*α*)) were purchased from Invitrogen (Mount Waverley, Victoria, Australia) and prepared for transfection according to the manufacturer's instructions. LNCaP and PC3 cells were transfected using Lipofectamine plus (Invitrogen) and Superfect (Qiagen, Doncaster, Victoria, Australia) respectively according to the manufacturer's instructions. Individual colonies surviving after 2–3 weeks selection were picked and propagated for analysis.

#### Confirmation of mRNA expression in INH*α*-transfected LNCaP and PC3 cell lines

Total RNA was extracted using RNeasy Mini Kit (Qiagen) according to the manufacturer's instructions. Reverse transcription (RT) was performed as previously described (Balanathan *et al*, 2004). *β*_2_-microglobulin (*β*_*2*_*mg*) was used as a housekeeping gene for block PCR. Primer sequences were: INHA, forward: CCTGTTCTTGGATGCCTTG; reverse: AGCTGGGCTGAAGTCACCT and *β*_2_ mg, forward: CCGTGTGAACCATGTGACTT; reverse: CAAACATGGAGACAGCACTC. Absolute quantitative real-time analysis was used to assess the levels of TGF*β*RIII mRNA expression in the clones. The analysis was performed on a LightCycler real-time PCR machine (Roche Diagnostic, Mannheim, Germany) using LightCycler Fast Start DNA Master SYBR Green 1 (Roche Diagnostic) according to the manufacturer's instructions. All experiments were carried out twice and duplicate readings were taken for each replicate. The quantity of mRNA was determined using a standard curve and all values were normalised using the housekeeping gene, hypoxanthine ribosyl transferase (*HPRT*). Primer sequences were: TGF*β*RIII, forward: TTCCCTGTTCACCCGACCTGAAAT; reverse: CGTCAGGAGGCACACATTA and HPRT, forward: TGTAATGACCAGTCAACAGGG; reverse: TGGCTTATATCCAACACTTCG.

#### Confirmation of protein expression by ELISA

Cell lysates and conditioned media (conditioned for 24 h) were prepared from EV- and INH*α*-transfected clones. Total protein (1 *μ*g *μ*l^−1^) was used for further analysis. Inhibin A and B and activin A concentrations were measured in triplicate using specific ELISA according to the manufacturer's instructions (Diagnostic Systems Laboratories, Webster, TX, USA). VEGF-A and VEGF-C ELISAs were measured in duplicate using specific ELISA according to the manufacturer's instructions (R&D Systems, Minneapolis, MN, USA). Two biological replicates were examined.

#### Direct cell counting: proliferation assay

LNCaP and PC3 cells were seeded at a density of 1 × 10^5^ cells per well and 5 × 10^3^ cells per well, respectively, in 24-well plates and incubated at 37°C. Triplicate wells were harvested by trypsinisation on days 1, 2, 3, 4 and 5, and numbers of cells per well were counted using haemocytometer. Each experiment was repeated twice. The results obtained from individual clones (EV and INH*α*) were pooled for each treatment.

#### Scratch wound assay: motility assay

Cells were plated in triplicate in 6- or 12-well plates and grown until approximately 70–80% confluence. The cell monolayer was then wounded and analysed over time as previously described (Sharp *et al*, 2004). Each experiment was repeated twice. The results obtained from individual clones (EV and INH*α*) were pooled for each treatment.

#### Intra-prostatic inoculation of LNCaP and PC3 cells

The experiments were in accordance with NHMRC of Australia guidelines. LNCaP (2 × 10^6^) or PC3 (5 × 10^5^) clones were injected orthotopically into the ventral lobe of the prostate gland (10 animals per clone) of male SCID mice as previously described (Zeng *et al*, 2006). After 7–9 weeks, mice were killed and primary prostate tumours were removed and weighed. In addition, regional lymph nodes were removed for analysis. Monoclonal human mitochondria antibody (1 : 100; Chemicon, Temecula, CA, USA) was used to determine the presence of human cells in the tumours as previously described (McCulloch *et al*, 2005). The monoclonal R1 antibody (7.5 *μ*g ml^−1^), kindly provided by Dr Nigel Groome, was used to determine INH*α* expression in tumours as previously described (Balanathan *et al*, 2004).

Lymph node volumes were determined using stereological analysis as previously described (McPherson *et al*, 2001). The lymph nodes were serially sectioned at 5 *μ*m thickness and using a random sampling scheme, every 20th section was chosen for analysis. Briefly, the computer program newCAST component (version 2.14; Visiopharm, Hørsholm, Denmark) was used to generate a point grid, and volumes of the lymph nodes were determined. Each section was examined using × 20 magnification and tissue sections were mapped to define tissue boundaries and were sampled at predetermined intervals along *x-* and *y*-axes using a single grid-counting frame. The volume was then determined using the equation=no of points for each tissue × area per point × distance; in this case the distance was defined by thickness of the sections (5 *μ*m) plus (5 *μ*m × 20 for every 20th section).

#### LVD in the intra-prostatic tumours

Lymphatic vessels were identified using lymphatic vascular endothelial hyaluronan receptor (LYVE-1), a marker of lymphatic endothelium (Banerji *et al*, 1999). Invasion of tumour cells into lymphatics was monitored by the presence of human mitochondrial protein-stained cancer cells in lymph vessels. Double immunostaining for LYVE-1 and mitochondria was performed on a Dako Autostainer (Dako, Glostrup, Denmark). The sections were incubated with LYVE-1 antibody (Fitzgerald, Boston, MA, USA) diluted at 0.5 *μ*g ml^−1^ for 2 h. LYVE-1 was detected by incubation with Envision polymer-anti-rabbit-HRP (Dako) for 15 min and visualised with diaminobenzidine (Dako). Sections were then incubated with Double Staining Enhancer (Zymed, San Francisco, CA, USA) for 15 min and exposed to mitochondrial antibody (Chemicon) diluted at 1 : 200 for 2 h. Secondary antibody, biotinylated rabbit anti-mouse IgG1 (Zymed) was applied and the immunoreactivity was detected by ExtrAvidin-Alkaline phosphatase (Sigma, St Louis, MO, USA) and visualised by reaction with Vector Red (Vector Laboratories, Burlingame, CA, USA). The sections were counterstained with hematoxylin (Dako) and immunolocalisation was examined using an Olympus BX-60 microscope (Tokyo, Japan).

Lymphatic vessels were counted using stereological methods as previously described (Balanathan *et al*, 2004). Lymphatic vessels were counted within tissue sections (of randomly selected INHA-positive prostate tumours, *n*=15 and EV tumours, *n*=11; using *n*=2 randomly selected sections per tumour) to assess the LVD within the tumour (intra-tumoural) region, the region in contact with both the tumour and the stroma (peritumoural) and the region away from tumour. LVD was expressed as the number of lymph vessels per mm^2^.

#### Statistical analyses

All statistical analyses were performed and results were analysed by ANOVA or *t*-tests as specified. The relationships between INH*α* expression and clinicopathological parameters were evaluated by Fisher's exact test. The mean staining intensity of patients positive for each of the respective clinicopathological parameter was compared to the mean staining intensity (reference) of those patients who were negative. The relative risks and 95% confidence intervals (CI) were estimated.

## Results

### Relationship between INH*α* expression and clinicopathological parameters in primary prostate adenocarcinomas

**Figure 1:**
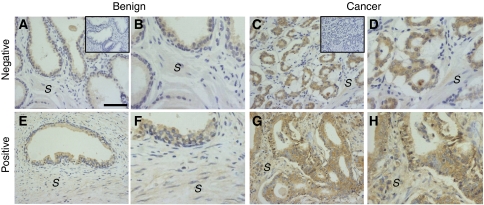
INH*α* expression in clinical specimens and its association to prostate disease. Immunohistochemical staining of INH*α* in primary prostate tumours from PCa patients with (**A**–**D**) organ-confined (negative) and (**E**–**H**) metastatic disease (positive). INH*α* immunostaining intensity in benign epithelial, cancer region (G3/G4) and stromal regions (*S*) is shown. Inset shows IgG control. Bar=200 *μ*m. **B**, **D**, **F** and **H** are enlargements of **A**, **C**, **E** and **G**, respectively.

Immunostaining revealed differential expression of INH*α* in benign epithelial, G3/G4 cancer regions as well as in the stroma of primary PCa tissues from patients with organ-confined disease (Figure 1A–D) and those with metastasis to the lymph nodes (Figure 1E–H). Association between clinicopathological prognostic factors and INH*α* expression is shown in Table 1. Elevated expression of INH*α* in the benign epithelial regions of the primary PCa tissues showed a higher relative risk in PCa patients positive for extracapsular spread (*P*=0.01). Similarly, elevated expression of INH*α* in the stroma of the primary PCa tissues showed a higher risk of PCa patients positive for extracapsular spread (*P*=0.0011), surgical margins (*P*=0.0006), VEGFR-3 expression (*P*=0.00067) and lymph node metastasis (*P*<0.0001). Further analysis showed that there was a significant increase in INH*α* staining in benign epithelial (*P*=0.018) and stromal (*P*<0.0001) regions but not in G3/G4 cancer regions in tissues from patients with lymph node metastasis compared to patients with organ-confined disease (data not shown).

### Isolation and characterisation of cells over-expressing INH*α*

The INH*α* expression profile in clinical specimens suggests pro-tumourigenic and pro-metastatic function for INH*α* in advanced PCa. Thus, to elucidate the effect of elevated levels of INH*α* expression on PCa cells, we stably transfected LNCaP and PC3 cell lines with an expression vector containing an INH*α* cDNA or with a control EV and confirmed the expression of the transgene by PCR (Supplementary Figure 1). Expression of dimeric proteins (inhibin A, inhibin B and activin A) was examined by ELISA. Analysis of cell lysates and conditioned media identified an increase in expression of inhibin B, but not inhibin A in INH*α*-transfected LNCaP cells and both inhibin A and inhibin B in the INH*α*-transfected PC3 cell lines (Table 2). There was no change in activin A level in the transfected cells. The expression of INH*α* protein was also validated by western blot and immunohistochemistry (data not shown). Immunohistochemistry of antibiotic selected clones revealed that the selected INH*α*-transfected clones were a heterogeneous population of INH*α*-positive and -negative cells. Because primary PCa cells are known for their heterogeneity and the aim of this study was to determine the effect of elevated levels of INH*α* in cancer, it was concluded the heterogeneous population was not going to bias the outcomes of the functional assays.

### Growth characteristics of INH*α*-transfected LNCaP and PC3 clones *in vitro*

**Figure 2:**
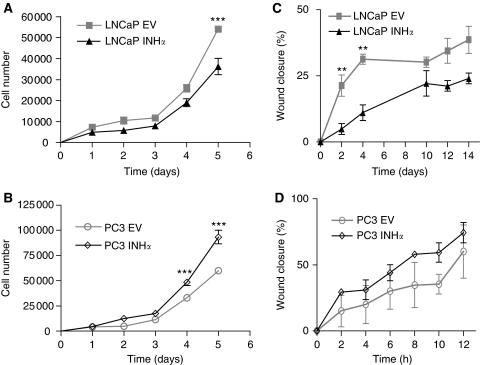
Effect of INH*α* over-expression on proliferation and motility of LNCaP and PC3 cells, *in vitro*. (**A** and **B**) Direct cell counts for EV- and INH*α* -transfected clones are shown; (**C** and **D**) the percentages of wound closure from EV- and INH*α* -transfected clones are shown. ^**^P<0.01, ^***^P<0.001 significant difference between the EV- and INH*α* -transfected clones. All results are from representative experiments. Data shown are mean±s.e. of the mean.

To evaluate the effect of over-expression of INH*α* on the growth of LNCaP and PC3 cell lines, we examined the proliferative capacity and motility of the various clones. Direct cell count demonstrated reduced proliferation of INH*α* over-expressing LNCaP cells (Figure 2A) and increased proliferation of PC3 cells (Figure 2B) when compared to their respective EV clones in the later stages of the growth curve. Monolayer wound healing assays showed INH*α* over-expressing LNCaP cells had a reduced rate of wound closure at earlier time points (Figure 2C) whereas INH*α* over-expressing PC3 cells demonstrated a trend towards increased rate of wound closure (Figure 2D). Because the significant changes in proliferation and motility observed in this study occur at different time points, it suggests that they are independent from each other.

### Effect of INH*α* over-expression on tumour growth and metastasis

**Figure 3:**
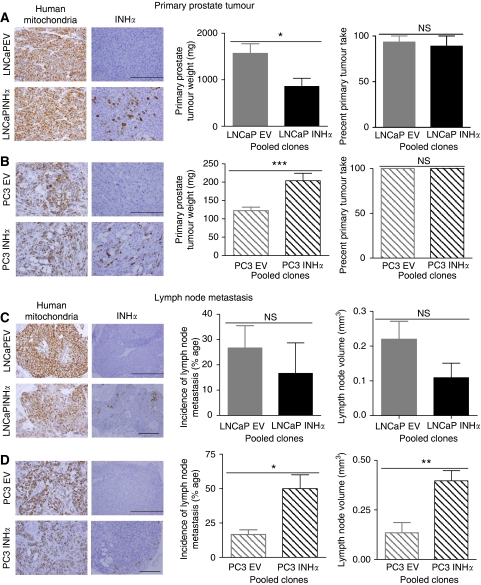
Effect of INH*α* over-expression on primary prostate tumour growth and lymph node metastasis. (**A**–**D**) Left, immunohistochemistry of primary prostate and lymph node tumours using human mitochondria and INH*α* staining confirmed the human origin of the cells in LNCaP- and PC3-inoculated mice and INH*α* expression in the tumours. Bar=200 and 500 *μ*m. (**A** and **B**) Primary prostate tumour weights (middle) and primary prostate tumour take (right). (**C** and **D**) Incidence of lymph node metastasis (middle) and lymph node volume (right). ^*^P<0.05, ^**^P<0.01, ^***^P<0.001 and no significant (NS) difference between the mean of the EV clones and the mean to the INH*α*-transfected clones. The bars represent EV-transfected LNCaP and PC3 clones in grey, INH*α*-transfected LNCaP and PC3 clones in black. Data are shown as mean±s.e. of the mean.

The influence of INH*α* on tumour growth and metastasis was determined *in vivo*. The clones were injected at the orthotopic site (prostate) and after 7–9 weeks primary prostate tumours as well as the adjacent lymph nodes were harvested. Primary prostate tumour size was determined and the incidence of lymph node metastasis was scored. Positive immunostaining for human mitochondrial protein confirmed that the primary and secondary tumours originated from intra-prostatic injection of human cells. INH*α* immunostaining was used to confirm INH*α* expression in tumours (Figure 3A–D, left). Immunostaining in the INH*α* tumours showed that INH*α* expression was not uniform within the tumour, which is consistent with a heterogeneous population of INH*α*-positive and -negative cells in the INH*α*-transfected clones we had observed previously. INH*α* immunostaining of the lymph nodes showed INH*α*-positive LNCaP cells to be distributed throughout the lymph node tumours whereas INH*α*-positive PC3 cells were confined to the outer tumour regions (Figure 3C and D, left). INH*α* over-expression in LNCaP cells did not affect orthotopic tumour take but significantly reduced the size of the primary tumour (*P*=0.0029) compared to the EV-transfected clones (Figure 3A, middle and right). Furthermore, INH*α* over-expression in LNCaP cells did not change the incidence of lymph node metastasis or the size of the lymph node tumours (Figure 3C, middle and right). INH*α* over-expression in PC3 cells had no effect on orthotopic tumour take but a significant increase in the primary prostate tumour size (*P*=0.005) was observed (Figure 3B, middle and right). INH*α* over-expression in PC3 significantly increased the incidence of lymph node tumours (*P*=0.0341) and lymph node tumour size (*P*=0.0047) compared to the EV-transfected clones (Figure 3D, middle and right). The altered tumour size following INH*α* over-expression may have influenced the formation of metastasis.

### LVD and invasion of tumour cells into lymphatics in INH*α* over-expressing primary prostate tumours

**Figure 4:**
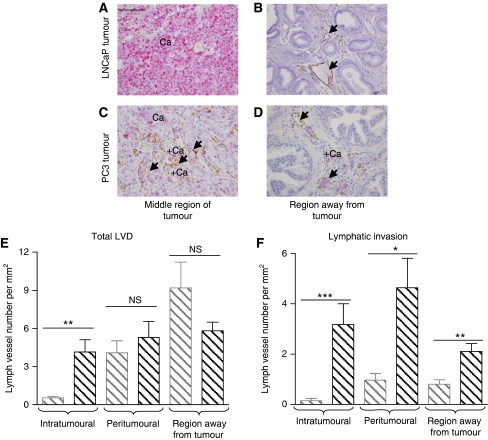
Effect of INH*α* on lymphatic vessel density and invasion. (**A**–**D**) Lymphatic vessels (LVs) were stained with LYVE-1 antibody (brown, ←) and human prostate cells (Ca) with human mitochondria antibody (purple). Bar=200 *μ*m. The total number of LVs (**E**) and LVs with cancer cells in their lumen (**F**) (for example, of such a vessel see ‘+Ca’ in panels **C** and **D**) in the intra-tumoural, peritumoural and region away from tumour of the primary prostate tumour were counted. ^*^P<0.05, ^**^P<0.01, ^***^P<0.001 and no significant (NS) difference between LVD in INH*α* over-expressing primary tumour compared to EV tumours. The bars represent EV-transfected PC3 clones in grey, INH*α*-transfected PC3 clones in black. Data are shown as mean±s.e. of the mean.

Changes to LVD and lymphangiogenesis are often associated with metastatic spread of cancer cells to the lymph nodes (Mattila *et al*, 2002; Zeng *et al*, 2005). To understand the mechanisms and to provide proof of metastatic spread observed in the mice injected with INH*α*-positive cells, we stained LNCaP and PC3 INH*α* and EV orthotopic tumours for LYVE-1, and human mitochondrial antibody to determine LVD and the degree of invasion of tumour cells into lymphatic vessels (lymphatic invasion) in the tissues. Consistent with our previous study (Zeng *et al*, 2006), the analysis of LNCaP tumours in the present study was complicated by the significantly larger size of the tumours compared to PC3 tumours. This larger size resulted in numerous necrotic areas in the LNCaP tumours, making it difficult to consistently define the different tumour peripheries and the intra- and inter-tumoural regions. For this reason stereological analysis of the LNCaP tumours was not possible. However, histological evaluation of the LNCaP tumours demonstrated no LYVE-1-positive lymphatic vessels within the tumour with lymphatic vessels only present in the regions away from the tumour (Figure 4A and B). In contrast, PC3 tumours had lymphatic vessels distributed throughout the tumour (Figure 4C and D). Stereological analysis of the PC3 tumours revealed a significant increase (*P*=0.0023) in total LVD in the intra-tumoural regions with no difference in LVD in peritumoural regions and regions away from tumour in INH*α*-positive tumours compared to the controls (Figure 4E). A significant increase in lymphatic invasion in the intra-tumoural (*P*=0.0002), peritumoural (*P*=0.0225) and regions away from tumour (*P*=0.0077) in INH*α*-positive tumour tissues compared to the controls was evident (Figure 4F).

### Factors inducing tumour growth and metastasis in INH*α* over-expressing PC3 tumours

The observed increase in LVD in INH*α*-positive PC3 tumours suggests that the metastatic spread of the cancer cells from the primary tumour site to the lymph nodes occurs through the process of lymphangiogenesis. Because VEGF-A and VEGF-C have been implicated in inducing/promoting metastasis to the lymph nodes in PCa (Karkkainen *et al*, 2002; Weis and Cheresh, 2005; Zeng *et al*, 2006), we went on to determine the expression of VEGF-A and VEGF-C proteins by ELISA (Table 3) in INH*α*- and EV-transfected clones *in vitro*. There was no significant change in the amount of VEGF-A and VEGF-C secreted by the different LNCaP clones. VEGF-C protein was significantly increased (*P*=0.0011) in the INH*α* over-expressing PC3 clones compared to their EV clones, however there was no significant change in secreted VEGF-A levels.

### Expression of TGF*β*RIII in LNCaP and PC3 transfected clones

**Figure 5:**
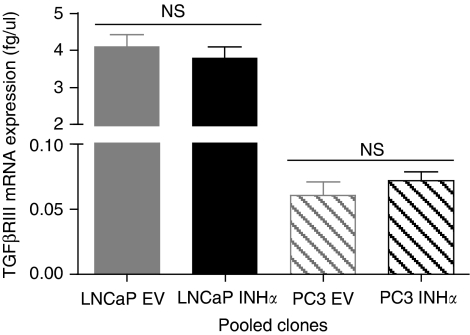
TGF*β*RIII expression in LNCaP and PC3 clones. Total RNA (2.5 *μ*g) was reverse transcribed (RT) and absolute quantitative real-time analysis was performed to determine the levels of TGF*β*RIII mRNA expression in the EV- and INH*α*-transfected clones. The bars represent EV-transfected LNCaP and PC3 clones in grey, INH*α*-transfected LNCaP and PC3 clones in black. Data are shown as mean±s.e. of the mean.

Although being a receptor for TGF*β*, TGF*β*RIII is also a receptor for inhibins (Wiater and Vale, 2003), and recent studies have reported loss of TGF*β*RIII as a common and important event in human PCa (Turley *et al*, 2007; Sharifi *et al*, 2007a). Down-regulation of TGF*β*RIII in PCa has been suggested to reflect loss of sensitivity to tumour suppressive inhibin by the PCa cells. To test this hypothesis, we determined the levels of TGF*β*RIII mRNA expression in the clones. Consistent with a recent study (Sharifi *et al*, 2007a), real-time analysis of PCa cell lines revealed LNCaP cells to have more TGF*β*RIII mRNA expression compared to PC3 cells. There was no change in TGF*β*RIII expression in INH*α*-transfected LNCaP or PC3 clones compared to EV-transfected clones (Figure 5).

## Discussion

Although inhibin is used as a biomarker for ovarian cancer, the biological function of elevated levels of INH*α* expression in PCa and certain types of ovarian cancer is unclear. This study is the first functional study to link up-regulation of INH*α* expression in androgen-independent prostate disease and progression of primary prostate and secondary tumours and metastasis.

We conducted a cross-sectional study to determine a link between INH*α* expression and a number of clinicopathological parameters: Gleason score, surgical margin status, extracapsular spread, lymph node status and VEGFR-3 expression, which are well-established prognostic factors of PCa (Wheeler *et al*, 1998; Cheng *et al*, 1999, 2001, 2005; Li *et al*, 2004). This study showed that an elevated level of INH*α* expression in the primary prostate tumour can be used as a predictive factor for prognosis in PCa. Univariate analysis showed a significant association between elevated levels of INH*α* in primary PCa tissues and extracapsular spread, surgical margins, VEGFR-3 expression and lymph node status. This finding is supported by our previous study that reported elevated levels of INH*α* in PCa patients to be associated with a higher risk of recurrence, although this association was not statistically significant (Risbridger *et al*, 2004b). The proposed cellular site for INH*α* expression and action is epithelial cells, however our immunostaining and analysis of the primary prostate tumour showed a significant association of elevated levels of INH*α* in both benign epithelial cells and stromal cells to extracapsular spread. There is increasing evidence that the surrounding microenvironment also has a major function in cancer cell growth, survival, invasion and metastatic progression, further supporting the pro-tumourigenic and pro-metastatic function of INH*α* in advanced PCa (reviewed in Chung *et al*, 2005; Alberti, 2006; Taylor and Risbridger, 2008). Likewise, Wheeler *et al* (1998) reported that invasion into or through the capsule of the prostate is strongly associated with both the ability of PCa to metastasise and with recurrence of cancer after radical prostatectomy. Our data link INH*α* expression in both epithelial and stromal cells with extracapsular spread, which suggests a role for INH*α* in advanced PCa. In addition, the observed up-regulation of INH*α* in benign epithelium and stromal regions in the primary prostate tumours in patients with lymph node metastasis suggests an association with VEGF-C-linked metastasis. Further analysis of our clinical data showed that INH*α* expression is stronger in benign epithelium and stroma but not the G3/G4 regions of the prostate in patients with lymph node metastasis compared to those with organ-confined disease. In PCa, Tsurusaki *et al* (1999) found that VEGF-C mRNA levels were significantly higher in lymph-node-positive tumours and that VEGFR-3-positive vessels were increased in the stroma of VEGF-C-positive tumours. Also, VEGF-C has been shown to promote growth of lymphatic vessels into and around tumours in animal models and this was associated with metastatic spread to the lymph nodes and sometimes to distant organs (Mandriota *et al*, 2001; Skobe *et al*, 2001; Mattila *et al*, 2002). Collectively, our analysis of INH*α* expression in primary prostate tumour tissues and the association to well-established prognostic factors strongly supports a pro-tumourigenic and pro-metastatic function for INH*α* in PCa.

Because the proposed cellular site for pro-tumourigenic and pro-metastatic action of INH*α* is cancer epithelial cells, we over-expressed INHA in metastatic epithelial PCa cell lines, LNCaP and PC3, to evaluate functionally the effect of elevated levels of INH*α* in PCa. A key event in the progression of PCa is the transition from an androgen-dependent to -independent stage, where subpopulations of tumour cells either gain resistance to, or adapt to, an androgen-depleted environment, and begin to proliferate resulting in the progression to highly aggressive and metastatic androgen-independent disease. Furthermore, when PCa is advanced or metastatic it is usually incurable and tends to metastasise to bone and lymph nodes. LNCaP cells, originally isolated from a lymph node metastasis (Horoszewicz *et al*, 1980, 1983), have retained their ability to respond to androgens, whereas PC3 cells, originally derived from a bone metastasis (Kaighn *et al*, 1979), are androgen independent. Thus these cell lines represent early and late stages of metastatic prostate disease.

INH*α* over-expression in LNCaP cells demonstrated reduced cell proliferation, migration and primary prostate tumour growth whereas INH*α* over-expression in these cells did not change the cells’ ability to metastasise to the lymph nodes nor did it influence the growth of the lymph node tumours. The lack of change in VEGF-C expression in INH*α*-transfected LNCaP cells is consistent with the unchanged metastatic ability of LNCaP cells *in vivo*. In contrast, INH*α* over-expression PC3 cells demonstrated increased cell proliferation, migration, primary prostate tumour and lymph node tumour growth. These cells showed increased metastasis to the lymph node, which was accompanied by an elevation of LVD and tumour cell invasion into lymphatics. These effects were associated with up-regulation of VEGF-C. Similarly, Karpanen *et al* (2001) reported that VEGF-C expression in a mouse tumour model strongly promoted the growth of tumour-associated lymphatic vessels, which in the tumour periphery were commonly infiltrated with the tumour cells. Although limited to the use of only two cell lines, the current study is the first to functionally show a pro-tumourigenic and pro-metastatic function for INH*α* in an androgen-independent model of metastatic prostate disease.

Another explanation for the different effects of INH*α* observed in the current study is the loss or gain of a yet-to-be-identified signalling pathway for INH*α* in either tumour suppression or promotion. We have shown that LNCaP cells express more TGF*β*RIII mRNA expression compared to PC3 cells and that the level of TGF*β*RIII mRNA expression is maintained after INH*α* over-expression. Loss of TGF*β*RIII during PCa progression has been suggested to be a reason for loss of sensitivity to the tumour suppressive effect of inhibin in prostate disease (Turley *et al*, 2007; Sharifi *et al*, 2007a, 2007b). Whether androgen status, different cell types and/or levels of TGF*β*RIII expression are responsible for the different effects of inhibin observed in the present study is an important area of future investigation.

Inhibins are known to be involved with regulating members of the TGF*β* superfamily, including TGF*β* itself, as well as activins and BMP, by competing for their receptors or co-receptors (Wiater and Vale, 2003; Farnworth *et al*, 2006, 2007). However, unlike other members of the TGF*β* superfamily (Pangas and Woodruff, 2000; Shi and Massagué, 2003), the signalling pathway(s) for inhibin have not yet been defined. Although it remains unclear how INH*α* mediates downstream cellular events leading to prostate disease, the observed effect of INH*α* in the present study suggests and supports an effect of inhibin which is independent of TGF*β*/activin in PCa. Owing to the presence of both *β*A and *β*B subunits in LNCaP and PC3 cells, the clones were able to produce dimeric inhibin A and inhibin B. The presence of inhibin B in both LNCaP and PC3 cells is significant as inhibin B is the pre-dominant form of inhibin in men and therefore more relevant to the study of PCa. Although the presence of inhibin A in PC3 may be of relevance to the observed phenotypes of PC3 cells, further investigation into the functional difference resulting from inhibin A *vs* inhibin B production in these cells was beyond the scope of this project. The level of inhibin A and inhibin B produced by the cells reflects the level of the *β*-subunits expressed and secreted by the respective cell lines (McPherson *et al*, 1999). Our study showed no change in the levels of activin A (*β*A*β*A), which indicates that observed effects are specific to inhibin and not a response to activin levels. Therefore, although TGF*β*/activins have been shown to induce expression of angiogenic/lymphangiogenic factors through P13K and/or SMAD2 pathways (Wagner *et al*, 2004; Kang, 2006) and TGF*β*/activins inhibit growth of PCa cells (Wilding *et al*, 1989; Wang *et al*, 1996; McPherson *et al*, 1997), the different responses of the PCa cell lines to elevated levels of INH*α* we have observed cannot be simply explained by abrogation of TGF*β*/activin signalling. Thus, the effect of inhibin on LNCaP cells observed in this study supports a role for inhibin in PCa independent of TGF*β*/activin. Also, the increased metastatic ability of INH*α* observed in INH*α* over-expressing PC3 cells cannot be due to antagonism of activin action because of the simultaneous expression of follistatin in these cells (McPherson *et al*, 1999). Follistatin is an activin-binding protein that binds to activin with a high affinity thereby inhibiting activin bioactivity (Phillips and de Kretser, 1998). Taken together, the current study demonstrates a direct action of INH*α* in PCa that is context and/or cell-type dependent.

In summary, this study showed both benign epithelial and stromal regions of primary prostate tumours to be the sites of INH*α* expression. We have also demonstrated a strong association of elevated levels of INH*α* to well-established prognostic factors of PCa. This finding has identified INH*α* as a very important predictive factor that can be used to identify patients at increased risk for disease progression and cancer death after radical prostatectomy, so that appropriate therapy can be selected. Finally, the current study has shown a pro-tumourigenic and pro-metastatic role for INH*α* in an androgen-independent model of metastatic prostate disease. This suggests that in the absence of androgens, elevated levels of inhibin may be driving the more aggressive and metastatic phenotypes of PCa tumour.

## Figures and Tables

**Table 1:** Relationships between the expression of INH*α* and clinicopathological parameters in prostate adenocarcinoma (*n*=37)

		**Benign regions**			**Cancer regions (G3/G4)**			**Stromal regions**		
**Parameters**	**No. of specimens**	**Mean intensity**	**Relative risk (95% CI)**	***P*-value**	**Mean intensity**	**Relative risk (95% CI)**	***P*-value**	**Mean intensity**	**Relative risk (95% CI)**	***P*-value**
*Combined Gleason grade*										
6	16	0.7	NA		1.88	NA		0.30	NA	
7	14	1.51	NA		2.21	NA		1.21	NA	
⩾8	7	1.29	NA		1.99	NA		2.00	NA	
										
*Extracapsular spread*										
Positive	24	1.43			2.13			1.41		
Negative	13	0.65 (reference)	2.07 (1.04–4.13)	0.01^a^	1.87 (reference)	1.27 (0.76–2.11)	NS^a^	0.08 (reference)	2.55 (1.39–4.65)	0.0011^a^
										
*Surgical margins*										
Positive	16	1.15			1.84			1.47		
Negative	21	1.12 (reference)	1.15 (0.68–3.08)	NS^a^	2.18 (reference)	0.71 (0.32–1.53)	NS^a^	0.50 (reference)	4.75 (1.62–13.93)	0.0006^a^
										
*VEGFR-3+ vessels*										
Positive	18	1.29			2.07			1.53		
Negative	19	0.97 (reference)	1.27 (0.62–2.60)	NS^a^	2.00 (reference)	1.20 (0.0–2.39)	NS^a^	0.33 (reference)	2.85 (1.28–6.37)	0.0067^a^
										
*Lymph node metastasis*										
Positive	16	1.41			2.06			1.93		
Negative	21	1.01 (reference)	1.62 (0.67–3.97)	NS^a^	2.00 (reference)	0.84 (0.48–1.46)	NS^a^	0.15 (reference)	13.22 (1.94–90.00)	<0.0001^a^

Abbreviations: VEGFR=vascular endothelial growth factor receptor; NA=not applicable; NS=not significant; CI=confidence interval.

a Fisher's exact test.

**Table 2:** Inhibin A, inhibin B and activin A protein expression in INH*α* clones

	**Cell lysates**			**Conditioned media**		
**Cell**	**Inhibin A**	**Inhibin B**	**Activin A**	**Inhibin A**	**Inhibin B**	**Activin A**
**lines**	**(pg ml^−1^)**	**(pg ml^−1^)**	**(ng ml^−1^)**	**(pg ml^−1^)**	**(pg ml^−1^)**	**(ng ml^−1^)**
*LNCaP*						
L16^a^	<10	166.3	<0.011	<10	<10	<0.011
L17^a^	<10	137.4	<0.011	<10	<10	<0.011
L18^a^	<10	118.5	<0.011	<10	<10	<0.011
L1^b^	<10	433.6	<0.011	<10	88	<0.011
L5^b^	<10	183.8	<0.011	<10	24.7	<0.011
L8^b^	<10	308.7	<0.011	<10	161.8	<0.011
						
*PC3*						
P128^a^	50.1	67.2	1.960	76	<10	8.860
P129^a^	56	64.1	1.780	80.8	<10	7.520
P130^a^	48.1	73	1.720	74.6	<10	8.820
P20^b^	228.1	149.7	1.280	197	185	7.320
P103^b^	194.1	186	1.690	132.3	226.4	9.340
P104^b^	221.6	187.8	1.670	194.9	177.3	11.320

a EV-transfected clones.

b INH*α* over-expressing clones.

**Table 3:** Effect of over-expressing INH*α* on VEGF-A and VEGF-C expression

	**LNCaP**		**PC3**	
	**EV clones**	**INH*α* clones**	**EV clones**	**INH*α* clones**
*VEGF-A*				
Secreted protein (pg ml^−1^)	1751±33.48	1747±26.02	1794±40.31	1712±34.64
				
*VEGF-C*				
Secreted protein (pg ml^−1^)	5.86±2.66	7.82±2.13	3377±566.0	6892±531.6^**^

Abbreviations: EV=empty vector; VEGF=vascular endothelial growth factor.

Data from individual clones were pooled and presented above.

^**^P=0.0011.
