# Supplementary material for: Elevated level of inhibin-α subunit is pro-tumourigenic and pro-metastatic and associated with extracapsular spread in advanced prostate cancer
Source: Br J Cancer. 2009 May 12;100(11):1784–93. doi: 10.1038/sj.bjc.6605089 (PMC2695696; doi:10.1038/sj.bjc.6605089)
Supplement: Supplementary Figure Legend [file 6605089x2.doc]

**Supplementary text**

**Results**

***Confirmation of mRNA expression in INHα-transfected LNCaP and PC3 cell lines***

The INHα-transfected LNCaP clones L1, L5 & L8 and the PC3 clones P20, P103 & P104 exhibited the expected 250bp DNA fragment amplified from the INHα transcript (Supplementary Figure 1). No transcript was expressed in EV-transfected LNCaP clones L16, L17, & L18 and PC3 clones P128, P129 & P130.

**Figure legend**

**Supplementary Figure 1** Characterization of EV- and INHα-transfected clones. Total RNA (2.5μg) was reverse-transcribed (RT). PCR performed revealed a 250bp PCR product in the INHα-transfected LNCaP **(A)** and PC3 **(B)** clones. Beta_2_microgloblin (*β_2_mg*) was used to confirm mRNA integrity and compare loading of the cDNA.
