# Supplementary material for: Elevated level of inhibin-α subunit is pro-tumourigenic and pro-metastatic and associated with extracapsular spread in advanced prostate cancer
Source: Br J Cancer. 2009 May 12;100(11):1784–93. doi: 10.1038/sj.bjc.6605089 (PMC2695696; doi:10.1038/sj.bjc.6605089)
Supplement: Supplementary file 2 [file 6605089x1.doc]

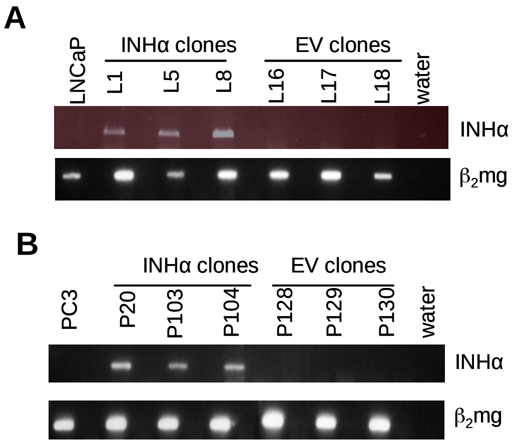
A
INHα clones
EV clones
LNCaP
L1
L5
L8
L16
L17
L18
water
INHα
β2mg
B
INHα clones
EV clones
PC3
P20
P103
P104
P128
P129
P130
water
INHα
β2mg
